# Interaction of melatonin and Bmal1 in the regulation of PI3K/AKT pathway components and cellular survival

**DOI:** 10.1038/s41598-019-55663-0

**Published:** 2019-12-13

**Authors:** Mustafa C. Beker, Berrak Caglayan, Ahmet B. Caglayan, Taha Kelestemur, Esra Yalcin, Aysun Caglayan, Ulkan Kilic, Ahmet T. Baykal, Russel J. Reiter, Ertugrul Kilic

**Affiliations:** 1 Regenerative and Restorative Medicine Research Center, Istanbul Medipol University, 34810 Istanbul, Turkey; 2 Department of Physiology, School of Medicine, Istanbul Medipol University, 34810 Istanbul, Turkey; 3 Department of Medical Biology, International School of Medicine, Istanbul Medipol University, 34810 Istanbul, Turkey; 4 Department of Medical Biology, School of Medicine, University of Health Sciences, 34668 Istanbul, Turkey; 5 Department of Medical Biochemistry, School of Medicine, Acibadem Mehmet Ali Aydinlar University, 34752 Istanbul, Turkey; 6 Department of Cell Systems and Anatomy, UT Health San Antonio, 78229 Texas, USA

**Keywords:** Cellular neuroscience, Circadian mechanisms, Stroke

## Abstract

The circadian rhythm is driven by a master clock within the suprachiasmatic nucleus which regulates the rhythmic secretion of melatonin. Bmal1 coordinates the rhythmic expression of transcriptome and regulates biological activities, involved in cell metabolism and aging. However, the role of Bmal1 in cellular- survival, signaling, its interaction with intracellular proteins, and how melatonin regulates its expression is largely unclear. Here we observed that melatonin increases the expression of Bmal1 and both melatonin and Bmal1 increase cellular survival after oxygen glucose deprivation (OGD) while the inhibition of Bmal1 resulted in the decreased cellular survival without affecting neuroprotective effects of melatonin. By using a planar surface immunoassay for PI3K/AKT signaling pathway components, we revealed that both melatonin and Bmal1 increased phosphorylation of AKT, ERK-1/2, PDK1, mTOR, PTEN, GSK-3αβ, and p70S6K. In contrast, inhibition of Bmal1 resulted in decreased phosphorylation of these proteins, which the effect of melatonin on these signaling molecules was not affected by the absence of Bmal1. Besides, the inhibition of PI3K/AKT decreased Bmal1 expression and the effect of melatonin on Bmal1 after both OGD *in vitro* and focal cerebral ischemia *in vivo*. Our data demonstrate that melatonin controls the expression of Bmal1 via PI3K/AKT signaling, and Bmal1 plays critical roles in cellular survival via activation of survival kinases.

## Introduction

Suprachiasmatic nucleus (SCN) of the hypothalamus regulates the rhythmic secretion of melatonin from the pineal gland, which in turn modulates the circadian rhythms and the sleeping pattern by directly controlling the circadian clock machinery through the ubiquitin-proteasome signaling pathway^1–4^. The endogenous circadian clock consists of self-sustained molecular clockwork which includes the transcriptional activators Bmal1 and Circadian Locomoter Output Cycles Kaput Protein (Clock), and transcriptional repressors Cryptochrome Circadian Regulator 1 (Cry1), Cryptochrome Circadian Regulator 2 (Cry2), Period 1 (Per1), and Period 2 (Per2). The Clock and Bmal1 heterodimer complex activates the transcription of both Per and Cry^5,6^. Thereby, activation of these core clock proteins regulates the molecular machinery of the circadian system based on the transcriptional/translational feedback loop, playing roles in metabolism, development and aging^7^.

Recently, high-throughput approaches demonstrated that melatonin plays crucial roles in signaling pathways either through its free scavenger activity, G-protein-coupled metabotropic receptor binding or receptor independent signaling. Although melatonin is a well-known neuroprotective molecule in conditions of brain injury, such as ischemic stroke, its exact mechanism has not been elucidated. However, it has been speculated that this neuroprotective effect could be receptor dependent^8,9^ or independent^10^.

In addition, rhythm proteins also play crucial roles in the pathophysiological processes including neurodegenerative disorders. There is strong evidence that the effects of Bmal1, which is responsible for the regulation and maintenance of circadian rhythms, are mediated through the survival kinases in neurodegenerative disorders, such as ischemic stroke^11^. Furthermore, a positive correlation was found between the core clock protein Bmal1 expression and neuronal survival, which were associated with the overactivation of survival kinases, such as phosphatidylinositol 3′-kinase/AKT (PI3K/AKT) and extracellular regulated kinase-1/2 (ERK-1/2) after ischemic stroke in mice^11^. These findings indicate that the role of melatonin in the physiological and pathophysiological processes are complex and not solely limited to its anti-oxidant properties^10,12^. Furthermore, the significance of downstream signaling molecules of melatonin, such as PI3K/AKT signaling pathways, was revealed after Alzheimer-, Parkinson diseases and ischemic-stroke^12–15^. However, the significance of Bmal1 protein and its relationship with intracellular signaling proteins, such as PI3K/AKT signaling pathway components are largely unknown. These findings indicate that Bmal1 protein might be one of the most critical target proteins of melatonin to understand the mechanisms of endogenous survival response to stroke. Targeted deletion of Bmal1 in mice or *D. melanogaster* leads to the loss of behavioral activities and increases reactive oxygen species which are involved in accelerated aging process^16,17^. It was also reported that the transcription factor Bmal1 may play an important role in neurodegenerative disorders in humans such as Parkinson disease, Huntington disease and Alzheimer disease^18,19^. In addition, the Bmal1 protein polymorphism is thought to increase the risk of Parkinson disease^20^. Notably, it was revealed that reduction of Bmal1 protein levels led to increased neuronal death in primary cell culture and in mice treated with a chemical inducer of oxidative injury and striatal neurodegeneration^21^.

In this context, here we investigated the relationship between melatonin and the core circadian-clock protein Bmal1 in detail. To exclude endogenous melatonin release and other effects of *in vivo* conditions, such as circulation and neuroendocrine interactions, we used cells, which were genetically modified, to define the role of Bmal1 in cellular survival and signaling. First, we investigated the effect of core clock protein Bmal1 alone or in combination with melatonin on cellular survival after oxygen glucose deprivation (OGD). Cellular survival in *in vitro* experiments consisted of OGD of Bmal1 overexpression or Bmal1 knockdown in mouse neuro2A (N2A) cells. Both groups were treated with 1 µM melatonin or vehicle at the beginning of the re-oxygenation. Then, the underlying signaling pathways in the presence or absence of Bmal1 and their response to melatonin were investigated after OGD. To investigate the effect of Bmal1 on intracellular signaling, co-immunoprecipitation coupled liquid chromatography tandem-mass spectrometry (LC-MS/MS) and planar surface immunoassay (PSI) were performed. In addition, how melatonin regulates the expression of Bmal1 was revealed by inhibition of PI3K/AKT signaling pathway both *vitro* after OGD and *in vivo* after focal cerebral ischemia.

## Resuls

### Melatonin increases Bmal1 protein expression

**Figure 1:**
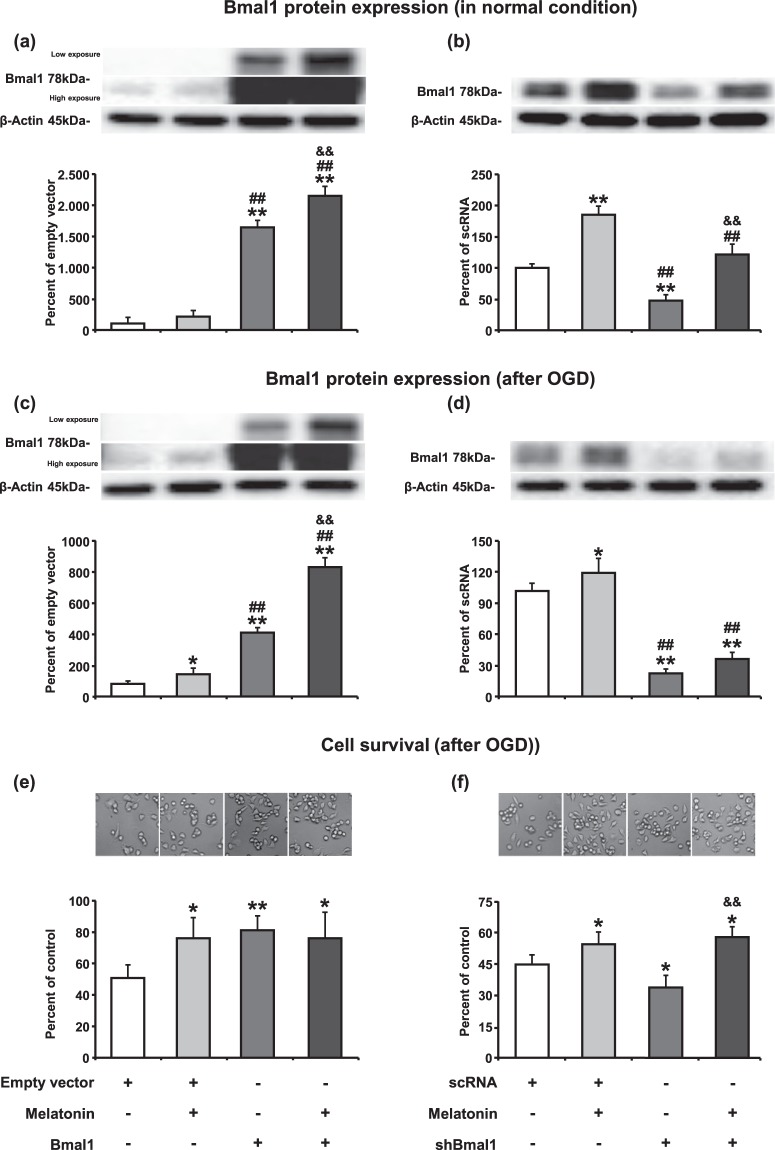
Melatonin increases Bmal1 protein expression in normoxia and after oxygen-glucose deprivation. Bmal1 protein expression both in the physiological conditions (**a**,**b**) and after OGD (**c**,**d**) was evaluated using Western blot. Cell survival analysis was performed 18 hours after OGD (**e**,**f**). 1 µM melatonin treatment or Bmal1 overexpression increases cell survival after OGD. The combination of melatonin and Bmal1 showed no cumulative effect on cell survival. β-actin was used as a loading control. Representative images of Western blot bands were given above their corresponding graphs. Presented data were cropped from full immunoblots for (**a**–**d**) shown in Supplementary Figs. S2–S5 and correspond to different exposure times. Data are mean ± S.D. values (three independent cell culture experiments were carried out (n = 3) and n = 3 blots/protein). **p < 0.01/*p < 0.05 compared with empty vector (pAcGFP1-N1)/scRNA ^##^p < 0.01compared with melatonin ^&&^p < 0.01 compared with Bmal1/shBmal1.

N2A cells were transfected Bmal1 overexpression plasmid or short hairpin RNA (shRNA) plasmid targeting Bmal1 (shBmal1) or their control plasmids (pAcGFP1-N1 for Bmal1 or scrambled RNA (scRNA) for shBmal1) using Lipofectamine 3000 reagent. To determine the transfection efficiency, forty-eight hours after transfection, fluorescence-activated cell sorting (FACS) analysis was performed. Results demonstrated that transfection efficiency achieved approximately 75–80 percent (Supplementary Fig. 1). The expression of Bmal1 protein upon transfection of appropriate plasmids was assessed by Western blot under physiological conditions. Results demonstrated that Bmal1 protein level was increased 16-fold in the overexpression group and was reduced by 60 percent in the shRNA group when compared with their appropriate controls (Fig. 1a,b). Administration of 1 µM melatonin resulted in significantly enhanced expression of Bmal1 protein in Bmal1 overexpressing or knockdown N2A cells under normoxic conditions (Fig. 1a,b) and after OGD (Fig. 1c,d).

### Both Bmal1 and melatonin increase cell survival after ***in vitro*** oxygen-glucose deprivation

To mimic focal cerebral ischemia *in vitro*, oxygen-glucose deprivation (OGD) method was used. After 6 hours of OGD, 18-hour-reperfusion was performed, and cell survival was analyzed. Significantly increased cell survival was observed in both Bmal1 overexpression and 1 µM melatonin administration alone groups. However, no cumulative effect on cell survival was observed when melatonin was given in the presence of Bmal1 overexpression (Fig. 1e). When Bmal1 expression was reduced with shRNA, survival rate after OGD was significantly decreased (Fig. 1f). Therefore, our results indicate that increase in Bmal1 protein level could enhance cell survival after OGD.

### Overexpression of Bmal1 enhances PI3K-AKT (Thr308), ERK-1/2 and mTOR signaling pathway

**Figure 2:**
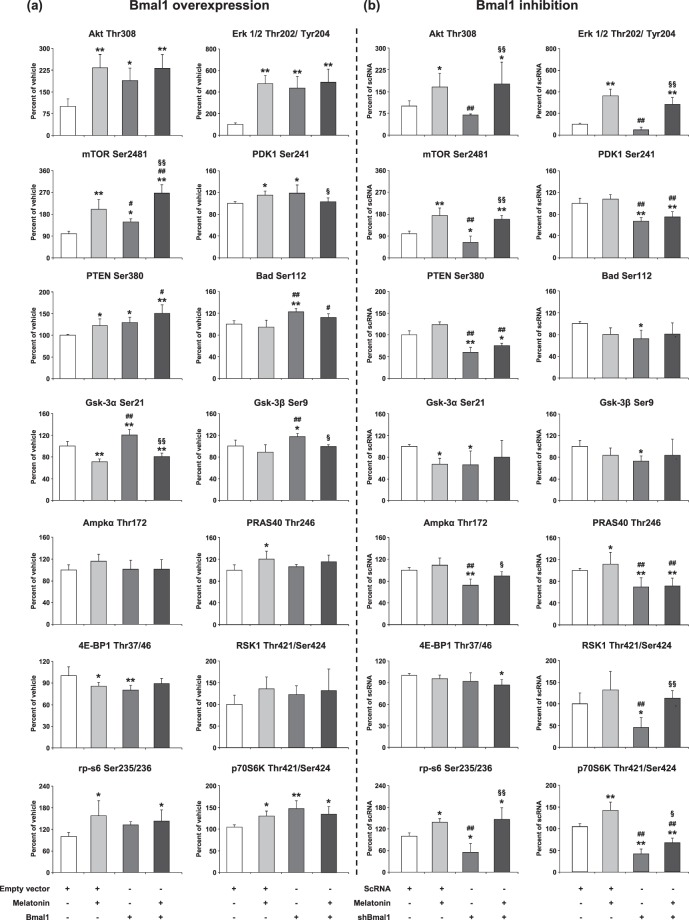
The role of Bmal1 overexpression/inhibition and melatonin in the regulation of AKT pathway components after OGD. To analyze PI3K signaling pathway components, cell extracts were prepared and analyzed using PathScan® AKT Signaling Antibody Array Kit. p-AKT (Thr308), p-ERK-1/2 (Thr202/Tyr204), p-mTOR (Ser2481), p-Pdk1 (Ser241), p-PTEN (Ser380), p-Bad (Ser112), p-Gsk-3α (Ser21), p-Gsk-3β (Ser9), (i), Ampkα (Thr172), p-PRAS40 (Thr246), p-4E-BP1 (Thr37/46), p-RSK1 (Thr421/Ser424), p-rp-s6 (Ser235/236), p-p70S6K (Thr421/Ser424) were evaluated for Bmal1 overexpression (**a**) and Bmal1 inhibition (**b**). Representative images of planar surface immunoassay analysis were given in the Supplementary Fig. S6. Data are mean ± SD (n = 4/group). **p < 0.01/*p < 0.05 compared with vehicle (empty vector; pAcGFP1-N1 Vector), ^##^p < 0.01/^#^p < 0.05 compared with Melatonin, ^§§^p < 0.01/^§^p < 0.05 compared with Bmal1 transfected group for (**a**) and **p < 0.01/*p < 0.05 compared scrambled RNA (scRNA), ^##^p < 0.01 compared with Melatonin, ^§§^p < 0.01/^§^p < 0.05 compared with shBmal1 group for (**b**).

To determine the underlying mechanisms responsible for the increase in cell survival in Bmal1 overexpression following OGD, we examined the expression level of PI3K/AKT signaling pathway which plays a pivotal role in cell survival and apoptosis (Fig. 2a,b). It is an intracellular signal transduction pathway that composes of a number of signaling molecules, including extracellular signal-regulated kinases (ERK-1/2), mechanistic target of rapamycin (mTOR), pyruvate dehydrogenase kinase-1 (PDK-1), phosphatase and tensin homolog (PTEN), Bcl-2-associated death promoter (Bad), glycogen synthase kinase-3α/β (GSK-3α/β), AMP-activated protein kinase α (AMPKα), The proline-rich AKT substrate of 40 kDa (PRAS40), eukaryotic translation initiation factor 4E-binding protein-1 (4E-BP1), 90 kDa ribosomal S6 kinase-1 (RSK1), ribosomal protein S6 (rp-s6) and Ribosomal protein S6 kinase beta-1 (p70S6K).

**Figure 3:**
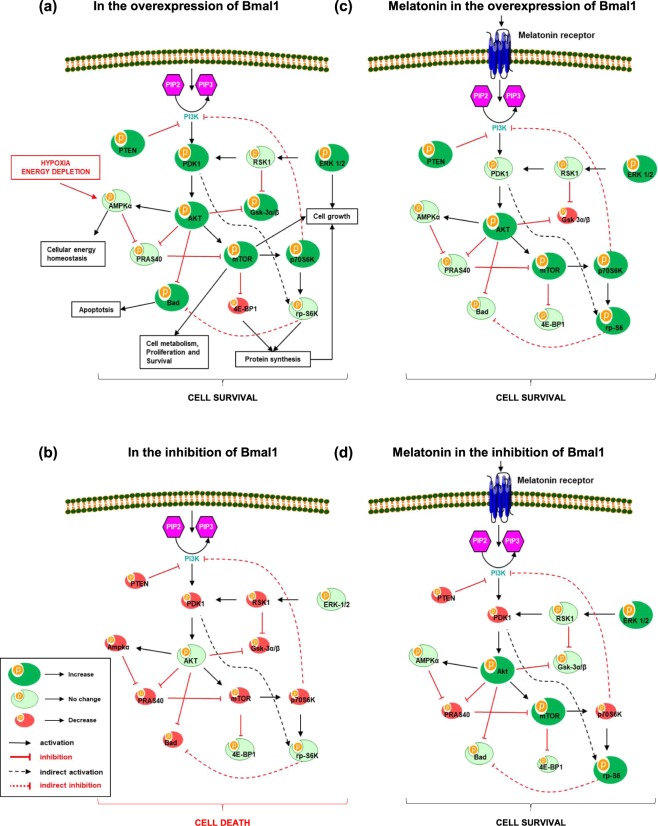
Regulation of PI3K/AKT signaling pathways. PI3K/AKT is an intracellular signal transduction pathway that is composed of a number of signaling molecules. AKT is activated via its upstream kinase PDK1, while PTEN is a major negative regulator of AKT signaling. When phosphorylated, inhibitory effect of PTEN on PI3K/AKT is decreased. Activated AKT stimulates mTOR phosphorylation, which is a central regulator of cell metabolism, growth, proliferation and survival. Both AKT, ERK-1/2 and mTOR are negative regulators of cell death (Kilic *et al*., 2017). Regulation of PI3K/AKT signaling pathways in the presence (**a**) and absence (**b**) of Bmal1, and contribution of melatonin in these conditions were also summarized (**c**,**d**).

We have also previously reported that circadian rhythms proteins especially Bmal1 overexpression is associated with the activation of PI3K/AKT signaling pathway, and may mediate neuronal survival following ischemic stroke^11^. Based on our previous studies, we speculated that Bmal1 could alter the IP3K/AKT signaling pathways after OGD. Increased Bmal1 protein expression enhanced AKT protein expression which was partially activated by phosphorylation of T308 by PDK1. It was observed that the increase of Bmal1 protein resulted in the decrease of 4E-BP1 protein and the increase of p70S6K protein expression which were responsible for dissociation of mTOR. In addition, incubation of Bmal1 overexpressed cells with melatonin synergistically increased mTOR protein expression. Moreover, increased levels of activated ERK-1/2, PTEN, GSK-3α/β and Bad were detected in Bmal1 overexpressed groups (Fig. 2a). However, in the knockdown of Bmal1 protein, several PI3K/AKT signaling components including Akt, Erk-1/2 and mTOR, were inhibited, which were reversed by melatonin (Fig. 2b). These results and particularly inhibition of PDK1, which is a core protein of PI3K/AKT pathway, indicate that PI3K/AKT pathway plays a crucial role in the neuroprotective effect of Bmal1. These results and the significance of PI3K/AKT pathway components were also summarized in a schematic diagram in Fig. 3.

### Identification and classification of Bmal1-related proteins using co-immunoprecipitation coupled LC-MS/MS analysis

**Figure 4:**
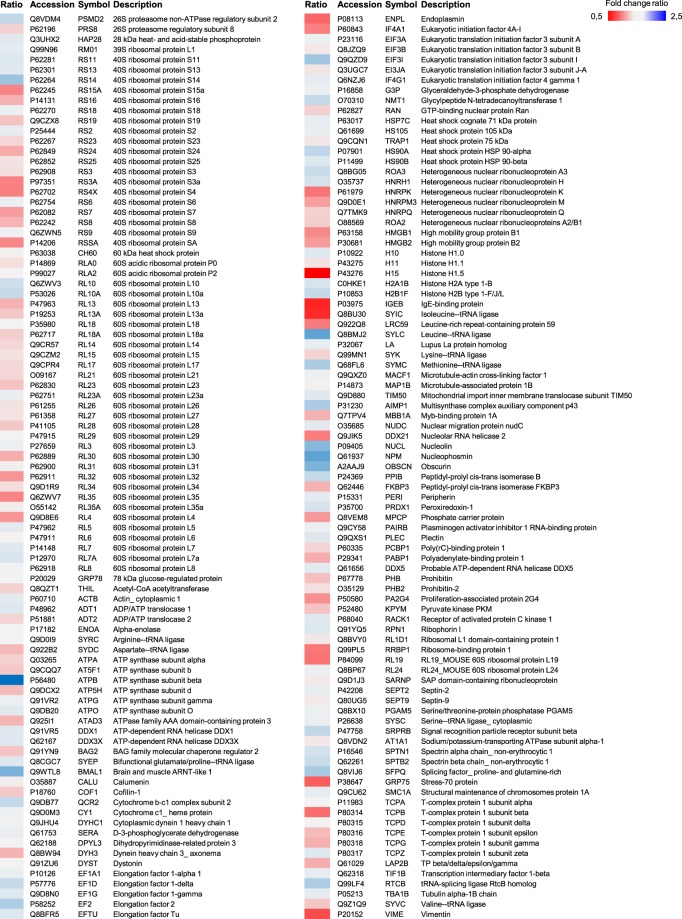
Identification of Bmal1 related proteins via co-IP coupled LC-MS/MS. After co-immunoprecipitation coupled liquid chromatogram tandem-mass spectrometry (LC-MS/MS) analysis, Bmal1 related proteins were identified. Comparisons (Bma1/Vehicle ratio) of differential protein expression were given by heat map analysis.

**Figure 5:**
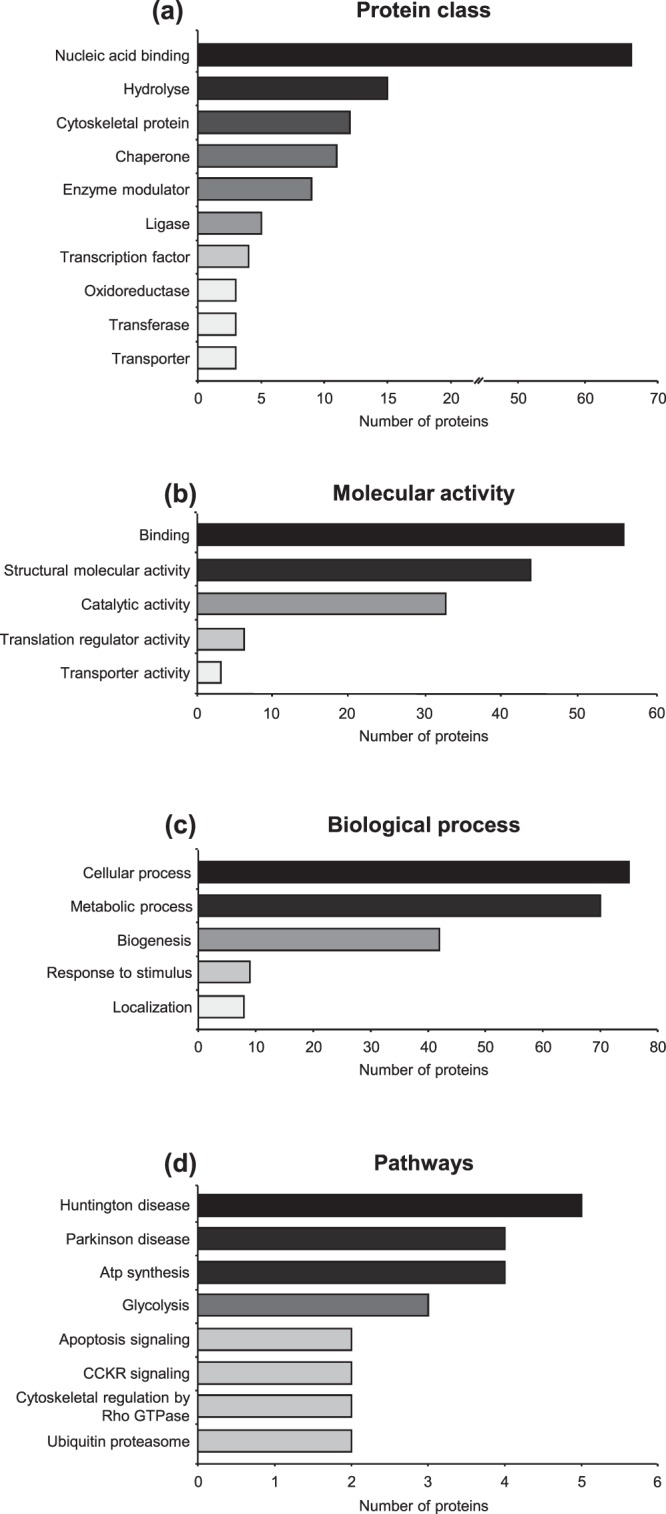
Classification of Bmal1 related proteins. Bmal1 related proteins were classified with respect to protein class (**a**), molecular activity (**b**), biological process (**c**), and pathways (**d**) via Protein ANalysis THrough Evolutionary Relationships (PANTHER, http://pantherdb.org) classification system.

A total of 178 proteins were detected after co-IP (with Bmal1 antibody) coupled LC-MS/MS analysis and listed in Fig. 4. All identified proteins were classified broadly into several categories according to the Protein ANalysis THrough Evolutionary Relationships (PANTHER, http://pantherdb.org) classification system (Fig. 5a–d). The identified proteins were predicted to belong to 10 different protein classes (Fig. 5a): 11 chaperone, 12 cytoskeletal protein, 9 enzyme modulator, 15 hydrolyse, 5 ligase, 67 nucleic acid binding, 3 oxidoreductase, 4 transcription factor, 3 transferase, and 3 transporter. According to the molecular activity-based classification, 55 binding, 43 structural molecular activity, 32 catalytic activity, 6 translation regulator activity, and 3 transporter activity proteins were identified (Fig. 5b). Moreover, these proteins were mapped to five major biological process (Fig. 5c). From the protein mapping, 75 cellular process, 70 metabolic process, 42 cellular component organization (biogenesis), 9 response to stimulus and 8 localization-related proteins were classified. Pathway analysis was performed according to the proteomic data (Fig. 5d). The top scored pathways were Huntington disease, Parkinson disease, ATP synthesis, glycolysis, apoptosis signaling, gastrin and cholecystokinin receptors mediated signaling map, cytoskeletal regulation by Rho GTPase and ubiquitin-proteasome pathway.

### Melatonin modulates Bmal1 regulation through PI3K/AKT dependent pathway after ***in vitro*** OGD

**Figure 6:**
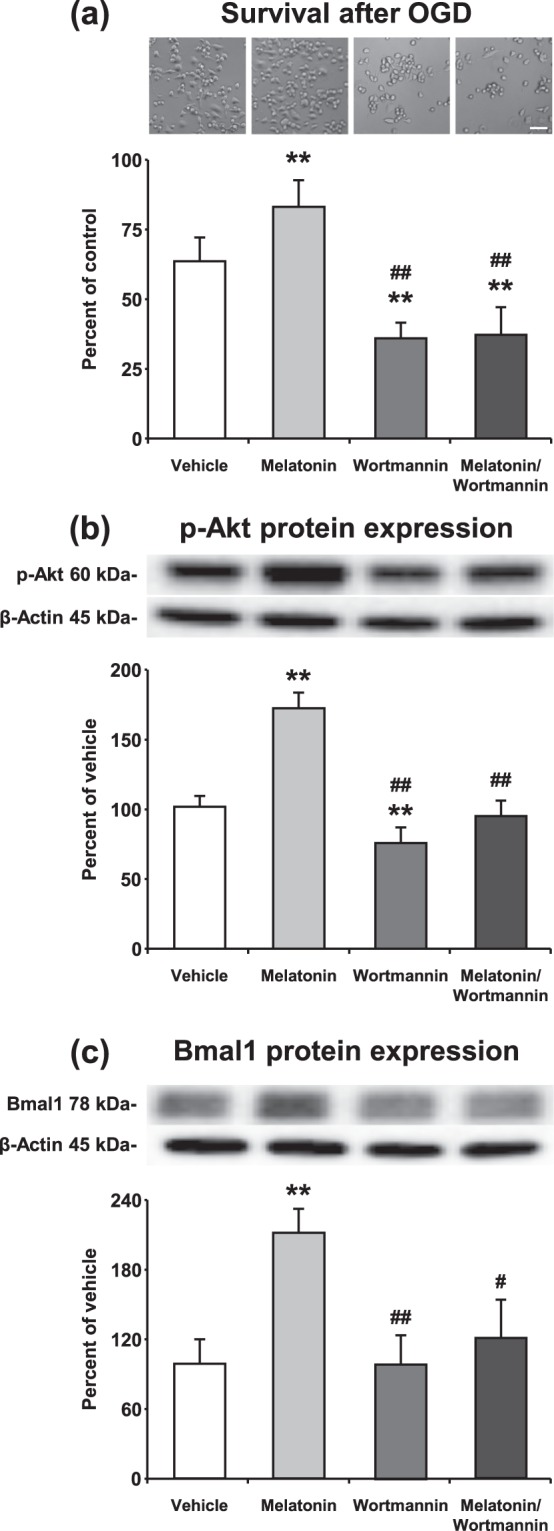
Inhibition of the PI3K signaling pathway prevents melatonin mediated Bmal1 expression after *in vitro* OGD. Blockage of PI3K signaling pathway by Wortmannin significantly reduced cell survival (the percentage of surviving cells was determined and normalized to the control group which was not subjected to OGD) and administration of 1 µm melatonin could not reverse its effect after OGD (**a**). p-AKT (**b**) and Bmal1 (**c**) levels were increased by melatonin administration and decreased by Wortmannin treatment. Representative images of Western blot bands were given above their corresponding graphs. β-actin was used as a loading control. Presented data were cropped from full immunoblots for (**b**,**c**) shown in Supplementary Figs. S7 and S8. Data are mean + S.D (three independent cell culture experiments were carried out (n = 3) and n = 3 blots/protein). **p < 0.01 compared with vehicle, ^##^p < 0.01/compared with melatonin group.

Phosphorylated AKT protein level was diminished 25–30 percent by 0.5 µM Wortmannin treatment. It was observed that inhibition of PI3K transduction pathway did not alter the Bmal1 expression, but significantly reduced cell survival after OGD (Fig. 6a). Conversely, administration of 1 µM melatonin after OGD increased p-AKT and Bmal1 protein expressions which led to an increase in cell survival. Correspondingly, AKT (Thr308) phosphorylation was significantly enhanced by melatonin and this increase in AKT phosphorylation was reversed by Wortmannin (Fig. 6b).

### Melatonin leads to an increase in Bmal1 protein expression after focal cerebral ischemia

**Figure 7:**
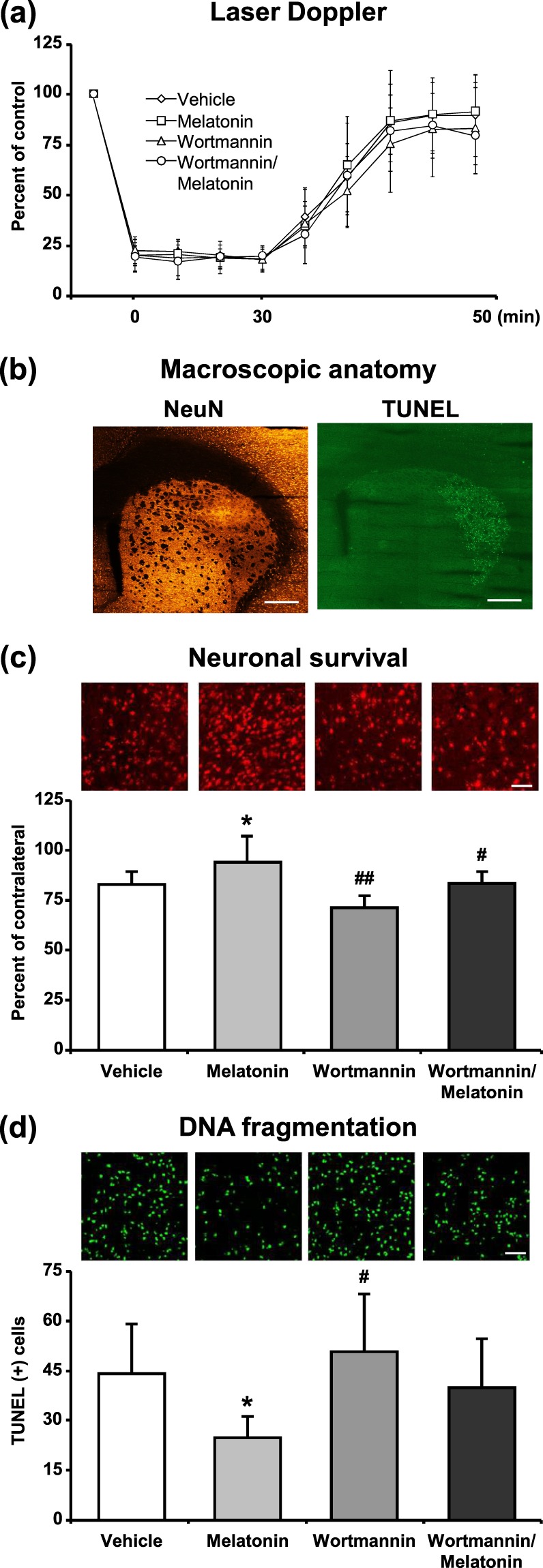
Melatonin decreased disseminate neuronal injury and increased neuronal survival after focal cerebral ischemia. Cerebral blood flow (CBF) was measured via laser Doppler flowmetry (LDF) during ischemia and at the onset of reperfusion (**a**). Neuronal survival (**c**) and disseminate neuronal injury (**d**) were analyzed 72 h after 30 min MCAO. Lower magnification images with anatomical landmarks for NeuN and TUNEL staining were given in (**b**). While melatonin decreased TUNEL (+) cells and increased neuronal survival, Wortmannin increased number of apoptotic cells and decreased neuronal survival rate after ischemia. Data are mean + S.D (n = 7 mice/group). *p < 0.05 compared with vehicle, ^##^p < 0.01/^#^p < 0.05 compared with melatonin group. Scale bars are 500 µm (**b**), 50 µm (**c**) and 100 µm (**d**).

**Figure 8:**
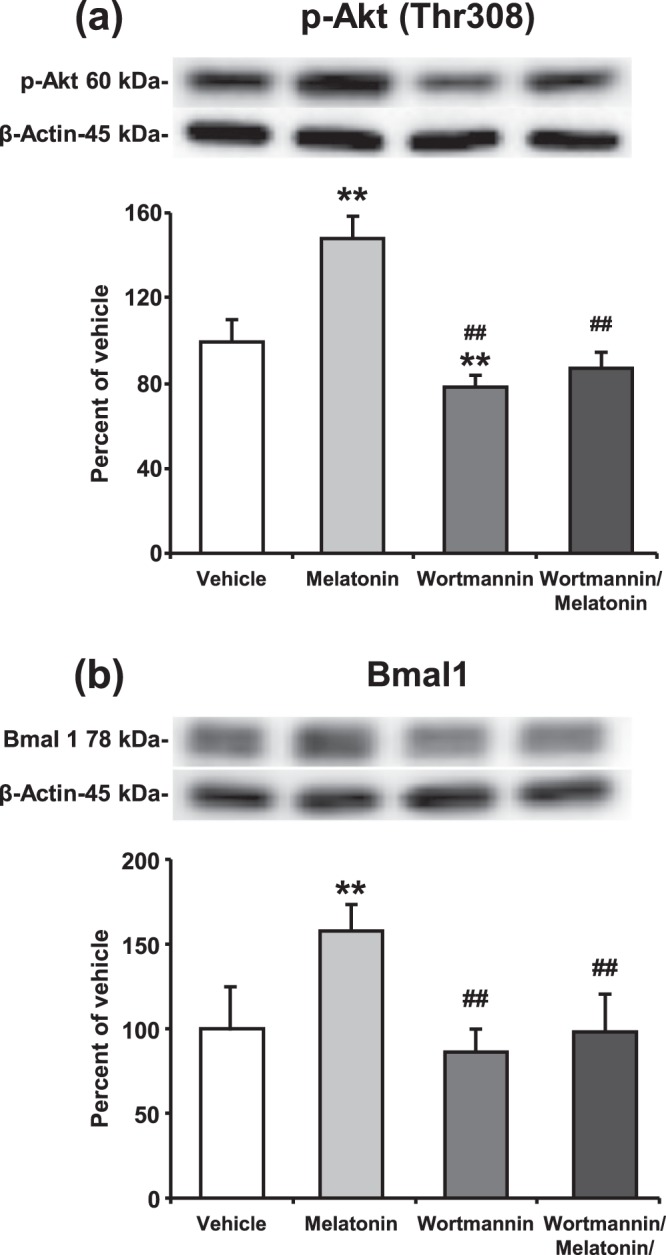
Melatonin increases the expression level of Bmal1 protein through PI3K/AKT signaling pathway after focal cerebral ischemia. Protein expression of (**a**) p-AKT (Thr308) and (**b**) Bmal1 were evaluated using Western blot from the brain tissues of mice submitted to 30 min MCAO. β-actin was used as a loading control. Representative images of Western blot analysis were given above their corresponding graphs. Presented data were cropped from full immunoblots for (**a**,**b**) shown in Supplementary Figs. S9 and S10. Data are mean ± S.D. values (n = 3 blots/protein). **p < 0.01 compared with vehicle, ^##^p < 0.01 compared with melatonin treated group.

Laser Doppler Flowmetry (LDF) was used to evaluate the changes in cerebral blood flow (CBF) during and after the onset of ischemia (Fig. 7a). An eighty five percent decrease in LDF values during the occlusion with a subsequent and rapid increase with the onset of reperfusion are routinely observed in our model of ischemic stroke^11,22^. Our data demonstrated that administration of 4 mg/kg melatonin increased neuronal survival and also reduced disseminate neuronal injury in the striatum after focal cerebral ischemia (Fig. 7c,d). It is well-known that especially 4 mg/kg melatonin increases phosphorylation of AKT (Thr308) after middle cerebral artery occlusion model in mice^12,23^. Similar to the results from *in vitro* OGD, melatonin increased p-AKT and Bmal1 protein expression and PI3K/AKT inhibitor Wortmannin diminished their levels (Fig. 8).

## Discussion

Circadian rhythms dependent on daily environmental variations regulate almost all physiological and behavioral activities within the 24-hour light/dark cycle. Activities of the biological clock are controlled by the master pacemaker located in the SCN of the anterior hypothalamus and by circadian oscillators in most peripheral tissues. At the cellular-molecular level, circadian rhythms are regulated and generated by a complex network of feedback loops which involves core clock genes, such as Clock, Bmal1, Cry1, Cry2, Per1, Per2, and Per3^5,6^. Circadian rhythms-controlled daily physiological activities such as body temperature, hormone secretion, blood pressure or energy metabolism may be involved in pathophysiological processes^17,24^. In addition, it has been revealed through several clinical studies, the circadian system also contributes to cellular injury response mechanisms after neurodegenerative disorders including Alzheimer disease, Parkinson disease, and focal cerebral ischemia^17,21,25^. In a previous study, we demonstrated for the first time that differential response to ischemic stroke occurs according to the time of the injury^11^.

Transcription factor Bmal1 and its binding partner Clock are the core components of the circadian machinery. Bmal1/Clock coordinates and regulates various cellular signaling pathways, such as metabolic pathways or signal transduction pathways^26,27^. Due to its essential contribution to the molecular signaling mechanisms after pathophysiological processes, Bmal1 is the most prominent protein among the circadian rhythms proteins. It was demonstrated that Bmal1 is important for the regulation of oxidative stress and DNA damage responses^28,29^. Yang *et al*. in a perspectives paper in Science Translational Medicine demonstrated that knockdown of transcription factor Bmal1 leads to loss of circadian timing which results in acceleration of aging and shortened lifespan in mice^30^. In addition, our previous study suggests that Bmal1 could strongly influence disseminate neuronal injury through AKT signaling pathway after focal cerebral ischemia^11^.

The pineal hormone melatonin regulates synchronization of the master pacemaker in the SCN which is directing all behavioral and physiological processes in a dusk and dawn manner. Previous studies in humans and rats presumed that indolamine melatonin directly influences its receptors in the SCN to control circadian timing^31,32^. However, it is a matter of debate, how melatonin acts on core clock proteins. Recent studies suggested that indolamine melatonin could regulate circadian rhythms proteins through the ubiquitin-proteasome pathway which inhibits the destruction of transcription factors^33,34^. It is predicted that inhibition of ubiquitin-proteasome pathway regulates Cry, Period and Bmal1 transcription factors (reviewed by Vriend and Reiter, 2015). Whereas the effect of Bmal1 on circadian rhythms machinery is well-documented, its role in cellular injury mechanisms is still largely unknown.

The purpose of the current study is to characterize in detail the impact of the Bmal1 on survival mechanisms after *in vitro* OGD. In addition to this, the relationship between Bmal1 and neuro-hormone melatonin was investigated in both physiological and ischemic conditions. Therefore, LC-MS/MS and PSI based broad-scale protein analyses were performed to investigate the effect of Bmal1 alone or combination with melatonin on intracellular signaling pathways. In this context, we have also examined the relationship between Bmal1, melatonin and PI3K signaling pathway using *in vivo* and *in vitro* ischemia.

Bmal1 protein expression was increased or inhibited in a murine Neuro2A cell line (N2A) using plasmid-mediated overexpression or shRNA mediated knockdown. Forty-eight hours after inducing overexpression/knockdown of Bmal1, we performed Western blot experiments to analyze Bmal1 protein expression. Plasmid-mediated overexpression significantly increased Bmal1 protein expression and lentiviral shRNA mediated knockdown significantly reduced Bmal1 protein expression. Both in physiological and ischemic conditions, 1 µM melatonin enhanced Bmal1 protein expression. This effect of melatonin on Bmal1 was also seen in the Bmal1 overexpressed cells. These findings support the previous studies which speculated that melatonin increases circadian rhythms protein Bmal1 in rodents^35,36^. Although Bmal1 expression was inhibited by shRNA, melatonin administration significantly increased Bmal1 protein level in both physiological and ischemic conditions. To explore the effect of Bmal1 on cell survival, OGD/R was performed which is an *in vitro* model that is widely used to mimic pathological changes of cerebral ischemia^37,38^. The overexpression of Bmal1 predominantly increased cell survival while lentivirus-mediated shRNA targeting Bmal1 exacerbated cell damage after *in vitro* OGD. One µM melatonin significantly improved cell survival but no synergistic effect was observed when melatonin was given to Bmal1 overexpressing N2A cells after OGD. While knockdown of Bmal1 protein expression decreased cell survival, this effect was reversed by the addition of melatonin.

To investigate the effect of Bmal1 on molecular signaling pathways in cell survival, comprehensive protein analyses were performed. Our detailed signaling pathway analyses demonstrated that members of the PI3K/AKT transduction pathway were under the regulation of Bmal1 protein. PI3K/AKT signaling pathway and its downstream proteins play an important role in cell proliferation, survival, apoptosis, protein synthesis, DNA repair, as well as other cellular functions. After OGD, Bmal1 increased cell survival through PI3K/AKT signaling pathway. Bmal1 regulates the activation of AKT phosphorylation at Thr308 which is activated via its upstream kinase PDK1. Up-regulation of AKT mediates multiple cellular processes such as cell proliferation, apoptosis, glucose metabolism, and neuronal injury mechanisms after pathophysiological conditions^39,40^. ERK-1/2 is a member of the mitogen-activated protein kinase (MAPK) which regulates a broad range of cellular functions such as proliferation, DNA fragmentation, and cell survival. In addition, it also controls cellular response to stress and influences several signal transduction pathways^41^. Bmal1 strongly increased phosphorylated Erk-1/2 protein level. The function of proapoptotic Bad protein is regulated by phosphorylation on either Ser112 or Ser136 residue^42^. It is important to note that melatonin slightly decreases phosphorylation of Bad, but Bmal1 significantly increases its phosphorylation. Bmal1 regulates phosphorylation of PTEN which increases GSK-3α/β activity by inhibiting PI3K/AKT signaling cascade. Importantly, Gsk3β specifically phosphorylates Bmal1, and primes it for ubiquitylation followed by proteasomal degradation^43^. Inhibition but not overactivation of Bmal1 attenuates phosphorylation of PRAS40 by AKT which relieves its inhibition of mTOR. Overactivation of mTOR which regulates several major cellular processes such as cell death, inflammation, and cell cycle, phosphorylates/activates rp-S6 and phosphorylates/inhibits the activity of 4EBP1. Taken together, our PSI results demonstrated that the modulation of Bmal1 protein regulates PI3K/AKT signaling cascade.

Our co-IP coupled LC-MS/MS analysis clearly demonstrated that, for the first time, 178 different proteins were in interaction with Bmal1. According to proteomic analysis, Bmal1 particularly interacted with nucleic acid binding proteins. Furthermore, it also interacted with hydrolyse, cytoskeletal, chaperone, enzyme modulator, ligase, oxidoreductase, transcription factor, transferase, and transporter proteins. Classification of identified proteins depending on molecular activity showed that Bmal1 interacted with binding, catalytic activity, structural molecular activity, translation regulator activity, and transporter activity related proteins. PANTHER classification based on biological process included 5 predominant groups; cellular process, metabolic process, cellular component organization or biogenesis, response to stimulus, and localization. In addition to this, 8 main Bmal1-related signaling pathways were identified as Huntington disease, Parkinson disease, ATP synthesis, apoptosis, CCKR signaling, cytoskeletal regulation, glycolysis, and ubiquitin-proteasome pathway.

Among the identified proteins, ten different proteins come into prominence. Class-III intermediate filament Vimentin (Vim) is an important protein which is responsible for filament dynamics. Furthermore, Zhang and colleagues suggest that continuous light causes post-translational modification of Vim and indolamine melatonin reverts the vimentin modification to the original form^44^. Histone H1.0 (H1f0) is a member of the H1 histone family of nuclear proteins which are a component of chromatin in eukaryotic cells^45^. Splicing factor, proline- and glutamine-rich (Sfpq) controls cell growth and regulates apoptosis-related genes^46^. Elongation factor 1-delta (Eef1d) is involved in eukaryotic protein synthesis and regulates cell cycle progression^47^. Spectraplakin family consists of two main members including dystonin (Dst) and microtubule-actin cross-linking factor 1 (Macf1). Dst is also known as Bullous pemphigoid antigen 1, and has a crucial role in anterior and retrograde protein transport^48^. Macf1 has various cellular functions such as cell proliferation, migration, signaling transduction and embryonic development^48,49^. Nucleolin (Ncl), contributes to cellular homeostasis mechanisms and has several cellular functions including ribosome biogenesis^48,50^. 39S ribosomal protein L1 (Mrpl1) is required for mitochondrial protein synthesis^51^. Nucleolar RNA helicase 2 (Ddx21), is an essential regulator for DNA polymerase I and II. Its activity is controlled by post-transcriptional regulatory mechanism which determines the activity of Ddx21, modulates genome dynamics, and safeguards genome integrity^52^. It was suggested that ATP-dependent RNA helicase DDX1 (Ddx1) plays an important role in NF‐κB transcriptional activity^53^.

It is well-documented that free melatonin is a radical scavenger molecule that prevents disseminate cell injury after *in vivo* and *in vitro* ischemia through PI3K/AKT signaling pathway^12,54,55^. Our study has clearly demonstrated that blockage of PI3K/AKT pathway by Wortmannin significantly reduced cell survival after *OGD in vitro* (Fig. 6a) and after ischemia *in vivo* (Fig. 7c). As expected, cell survival was significantly increased with melatonin-mediated activation of PI3K/AKT signaling pathway. While administration of melatonin significantly increased the level of Bmal1 protein, inhibition of the PI3K/AKT signaling cascade with Wortmannin was shown to inhibit the protein expression of Bmal1 slightly. When the AKT signaling pathway is blocked by Wortmannin, melatonin could not display any effect on neither cell survival nor Bmal1 protein expression after ischemia.

Based on the findings of this study, Bmal1 increases cellular survival after oxygen-glucose deprivation in N2A cells, and this was associated with increased expression of AKT, ERK-1/2 and mTOR survival pathways. More profoundly, we demonstrated that neurohormone melatonin regulates Bmal1 protein expression in physiological conditions and after OGD via the activation of PI3K/AKT signaling pathways. In addition, Bmal1-interacting proteins, which were identified using co-IP coupled LC-MS/MS analysis, suggested the interplay of several other proteins; playing roles in neurodegenerative diseases, ATP synthesis, apoptosis and glycolysis. Also, we have not observed synergistic effects of melatonin and Bmal1 with the exception of mTOR activation, which needs to be investigated further in future studies.

## Materials and Methods

### Ethics statement for animal experiments

*In vivo* part of this study has been conducted in accordance with the ethical standards according to the Declaration of Helsinki and according to national and international guidelines and has been approved by the Ethics Committee of Istanbul Medipol University (Reference number: 23/02/2018–10). All animals were maintained under a constant 12-h light/dark cycle (lights on at 06:00 daily).

### Cell culture

Mouse N2A cells were purchased from American Type Culture Collection (ATCC). N2A cells were maintained in Dulbecco’s modified Eagle’s medium (DMEM), low glucose, GlutaMAX^TM^, pyruvate (21885025, Thermo Fisher) supplemented with 10 percent fetal bovine serum (FBS; P30-1985, Pan Biotech) and 100 U/ml penicillin-streptomycin (P0607300, PAN Biotech) at 37 °C in a humidified atmosphere of 95 percent air and 5 percent CO_2_.

### Cloning experiments

Total RNA from N2A cell cultures was isolated using AllPrep DNA/RNA/Protein Mini Kit (80004, Qiagen) in accordance with the manufacturer’s protocol. To obtain complementary DNA (cDNA), DNA was synthesized from the RNA template using Transcriptor First Strand cDNA Synthesis Kit (04896866001, Roche). The coding sequence of Bmal1 (NCBI ref. seq. NM_007489.4) was amplified by using primers (Forward: 5′-AGTCAGTCGACAATGGCGG ACCAGAGAATGG-3′ and reverse 5′-AGTCAGGATCCAACAGCGGCCATGGCAAGT-3′) with fast digest restriction enzymes SalI (FD0644, Thermo Fisher) and BamHI (FD0054, Thermo Fisher). PCR product and pAcGFP1-N1 Vector (632469, ClonTech) were digested by using SalI and BamHI restriction enzymes. Next, they are ligated using T4 DNA ligase (EL0014, Thermo Fisher). Thereafter, insert was confirmed by sequencing. For Bmal1 knockdown, Smartvector Lentiviral Mouse Arntl mCMV-TurboRFP (targeted sequences: 5′-AGCCATTGCTGCCTCATCG-3′, 5′-GACATGAAGTCGCTGATGG-3′, and 5′-CAAATTTCCCATCTATTGC-3′) and SMARTvector non-targeting mCMV-TurboRFP were purchased from Dharmacon.

Five ×10^5^ cell/well were seeded on 6-well cell culture plates (3516, Corning). Next day, transfection was performed using Lipofectamine 3000 (L3000015, Thermo Fisher Scientific) as described by the manufacturer’s protocol. Briefly, 2.5 µg DNA and 5 µl Lipofectamine^TM^ 3000 reagent and 5 µl P3000 reagent were mixed in 250 µl Opti-MEM. After 10 min incubation at room temperature, the DNA-lipid complex was added to cells. Six hours after transfection, the medium was replaced with fresh DMEM incubated at 37 °C in a moist atmosphere containing 5 percent CO_2_. Forty-eight hours after transfection, OGD was performed.

### Fluorescence-activated cell sorting (FACS)

To determine transfection efficiency cells were isolated from cell culture dish and rapidly sorted utilizing a high-speed cell sorter (BD Influx cell sorter, Becton Dickinson, New Jersey, USA). green fluorescent protein (GFP) positive cells for Bmal1 overexpression or red fluorescent protein (RFP) positive cells for shBmal1 were analyzed and collected.

### Oxygen-Glucose deprivation (OGD)

To mimic *in-vivo* ischemia, OGD was performed 48 hours after transfection. For OGD, normal culture medium was replaced with equilibrated (exposed 5% CO_2_, 95%N_2_) no-glucose Dulbecco’s Modified Eagle Medium (DMEM; 11966, Gibco). Then, plates were transferred into a hypoxia incubator chamber (27310, Stemcell Technologies) supplemented with a gas mixture composed of 1 percent O_2_, 5 percent CO_2_, 94 percent N_2_. After six hours of incubation at 37 °C, OGD medium was replaced with fresh DMEM supplemented with 10 percent FBS and cells were incubated for 18 hours for re-oxygenation in an incubator maintained in a 5 percent CO_2_ atmosphere at 37 °C. At the end of the reperfusion, cells were harvested for protein analysis experiments.

### Cell survival analysis

Immediately before inducing OGD, number of cells were counted from nine different regions of interest (ROI). Then, six-hour OGD followed by eighteen-hour reperfusion was performed. At the beginning of the reoxygenation, plates were washed once with no-glucose DMEM to remove dead and unattached cells. At the end of the reperfusion, cells were counted from nine different ROIs again. Eventually, the percentage of surviving cells was determined and normalized to the control group which was not subjected to OGD. During *in vitro* experiments all cell counting was performed in a blinded manner.

### Western blot

Cell lysates or brain tissue samples harvested from the ischemic striatum of mice exposed to 30 min middle cerebral artery occlusion (MCAO) were pooled, homogenized and treated with Protease/Phosphatase inhibitor cocktail (5872, Cell Signaling). Protein concentration was determined via using Qubit 3.0 Fluorometer (Q33216, Invitrogen, Life Technologies Corporation, Carlsbad, CA, USA) according to the manufacturer’s protocol. Twenty micrograms of protein were size-fractionated using 4–20% Mini-PROTEAN TGX (4561096, Bio-Rad, Life Sciences Research) gel electrophoresis and then transferred to a PVDF membrane using the Trans-Blot TurboTransfer System (1704155, Bio-Rad, Life Sciences Research). Thereafter, membranes were blocked in 5% nonfat milk in 50 mMol Tris-buffered saline (TBS) containing 0.1% Tween (TBS-T; blocking solution) for 1 h at room temperature, washed in 50 mMol TBS-T, and incubated overnight with monoclonal rabbit Bmal1 (14020; Cell Signaling) or polyclonal rabbit phospho-AKT (Thr308) (9275, Cell Signaling). Following day, blots were washed with 50 mM TBS-T and incubated with horseradish peroxidase-linked goat-anti-rabbit (sc-2004; Santa Cruz Biotechnology) antibody (diluted 1:2500) for 1 h at room temperature. All of the proteins to be examined were studied as triplicate. To control protein loading PVDF membranes were stripped and re-probed with polyclonal rabbit anti-β-actin antibody (4967; Cell Signaling Technology). PVDF membranes were developed via Clarity Western ECL Substrate kit (1705060, Bio-Rad; Life Sciences Research) and visualized using the ChemiDoc MP System (1708280, Bio-Rad; Life Sciences Research). Expression level of proteins were analyzed densitometrically using an image analysis system (Image J; National Institute of Health, Bethesda, MD, USA), corrected with values determined on β-actin blots.

### Planar surface immunoassay (PSI)

As previously described^11^, PI3K/AKT transduction pathway was analyzed using planar surface immunoassay (PSI), (Pathscan®, 9474; Cell Signaling) which allows for the detection of p-AKT (Thr308), p-ERK-1/2 (Thr202/Tyr204), p-mTOR (Ser2481), p-Pdk1 (Ser241), p-PTEN (Ser380), p-Bad (Ser112), p-Gsk-3α (Ser21), p-Gsk-3β (Ser9), p-Ampkα (Thr172), p-PRAS40 (Thr246), p-4E-BP1 (Thr37/46), p-RSK1 (Thr421/Ser424), p-rp-s6 (Ser235/236), p-p70S6K (Thr421/Ser424). Briefly, 100 µl array blocking buffer was added onto the membrane and incubated for 15 min at RT on an orbital shaker. Then, equal amounts of protein (75 µg) was loaded and incubated for overnight at 4 °C on an orbital shaker. Following day, membrane was washed with array wash buffer. Afterward, 75 µl detection antibody cocktail was added to each well and incubated for 1 h at RT on an orbital shaker. Next, membrane was washed and incubated for 30 min with horseradish peroxidase-linked streptavidin at RT. At the end of the incubation membrane was covered with LumiGLO/Peroxide reagent and visualized using the ChemiDoc MP System (Bio-Rad; Life Sciences Research). Expression level of 16 phosphorylated proteins predominantly belonging to the AKT signaling pathways were analyzed densitometrically using an image analysis system (Image J; National Institute of Health, Bethesda, MD, USA). Protein levels were calculated and corrected with respect to negative and positive controls on array slides.

### Immunoprecipitation

One hundred fifty μg of cell lysate in 200 μl lysis buffer supplemented with protease and phosphatase inhibitor cocktail was incubated with 100 μl Protein G magnetic beads (LSKMAGG02, Millipore) for 20 min at RT for sample pre-clearing. Four μg of Bmal1 antibody was diluted 1:25 in PBS (pH 8) and incubated with 100 μl magnetic beads for 30 min at 4 °C on a rotator, washed three times with PBS (pH 8) and rotated for 45 min at RT with 1 ml DMP (D8388, Sigma) buffer (6.5 mg DMP in 1 ml 0.2 M triethanolamine, pH 8 (90279 Sigma)). Beads were washed and rotated for 1 h at RT in blocking buffer (0.1 M ethanolamine pH 8 (9508 Sigma). Beads were then washed once with PBS, pH 7.4 and put on a rotator with pre-cleared cell lysates overnight at 4 °C. On the next day, beads were washed twice with 1 ml suspension buffer (0.1 percent Tween-20, 0.02 percent sodium azide in PBS, pH7.4), precipitates were eluted with 50 μl elution buffer (0.1 M Glycine HCl, pH 2.5) and neutralized in 1:1 volume of Tris-HCl, pH 8.

### Sample preparation for liquid chromatography tandem-mass spectrometry (LC-MS/MS) analysis

Sample preparation was performed as previously described^2,11^. Tryptic peptides were generated according to the Filter Aided Sample Preparation Protocol (FASP). The brain tissues were taken from ischemic striatum and were homogenized in 50 mM ammonium bicarbonate and lysed by heating at 95 °C in UPX buffer (Expedeon). After incubation at 4 °C for an hour, samples were centrifuged at 14,000 × g for 10 min and the supernatants were transferred to a clean 1.5 ml microcentrifuge tube. The total protein concentration was measured with the Qubit assay. Tryptic peptides were generated by FASP kit (Expedeon). Briefly, 50 µg protein was filtered with 6 M urea in a 30 kDa cut-off spin column, alkylated with 10 mM iodooacetamide in the dark for 20 min at room temperature and incubated with trypsin (1:100 trypsin to protein ratio, Pierce) overnight at 37 °C. The tryptic peptides were eluted from the columns and lyophilized. The peptides were dissolved in 0.1 percent formic acid (FA, Sigma-Fluka) and diluted to 100 ng/μl before injecting to the LC-MS/MS system.

### LC-MS/MS analysis and data processing

The LC-MS/MS analysis and the subsequent protein identifications were done according to a previously published protocol^2,11,56,57^. Briefly, the tryptic peptides were loaded onto the ACQUITY UPLC M-Class coupled to a SYNAPT G2-Si high definition mass spectrometer (Waters). The columns were equilibrated with 97% mobile phase A (0.1% FA in UHPLC grade water, Merck) and temperature was set to 55 °C. Peptides were separated by a 90 min gradient elution from the trap column (Symmetry C18, 5 μm, 180 μm i.d. × 20 mm, Waters) to the analytic column (CSH C18, 1.7 μm, 75 μm i.d. × 250 mm, Waters) at 0.400 μl/min flow rate with a gradient from 4% to 40% ACN containing 0.1% FA (v/v). Positive ion modes of MS and MS/MS scans with 0.7 sec cycle time were performed sequentially. 10 V was set as low collision energy and 30 V as high CE. The ions were separated by ion mobility separation (IMS). A wave velocity was ramped from 1000 m/s to 55 m/s over the full IMS cycle. The release time for mobility trapping was set as 500 μs, trap height was set to 15 V. IMS wave delay was 1000 μs for the mobility separation after trap release^58^. Without any precursor ion preselection, all the ions within 50–1900 m/z range were fragmented in resolution mode. Additionally, 100 fmol/μl Glu-1-fibrinopeptide B was infused as lockmass reference with a 60 s interval. To identify and quantify the peptides, Progenesis-QI for proteomics software (Waters) was used. All proteins were identified by at least 3 unique peptide sequences and then, expression ratio of proteins was calculated.

### Inhibition of phosphatidylinositol 3-kinase/AKT signaling pathway ***in vitro*** and ***in vivo***

To inhibit PI3K/AKT signaling pathway, Wortmannin (Sigma Aldrich) was used for *in vitro* or *in vivo* ischemia. For cell culture experiments, vehicle (100 percent Dimethyl sulfoxide (DMSO) in 1 µl) or 0.5 µM Wortmannin (dissolved in 1 µl 100 percent DMSO) was performed 30 min before OGD.

For animal experiments, mice were anesthetized with 1% isoflurane (30% O_2_, reminder N_2_O) and placed into a stereotaxic device (World Precision Instruments). After a small midline scalp incision, vehicle (100 percent DMSO in 2 µl) or Wortmannin (0.1 mM in 2 µl of 100 percent DMSO) was carefully injected intrastriatally (2.5 mm lateral to the sagittal suture, 0.4 mm anterior to the bregma, and 3.5 mm deep) within 5 minutes. 30 min after the delivery of vehicle or Wortmannin, MCAO was performed^12^.

### Animal experiments

A total 28, 8–12 weeks male C57BL6/J mice (Jackson Laboratory, Sacramento, CA and Bar Harbor, ME) were randomly assigned to one of four groups and treated with intraperitoneal (i.p.) delivery of (i) vehicle (50 μl isotonic saline/5% ethanol; n = 7), (ii) melatonin (4 mg/kg, dissolved in 0.9% isotonic saline/5% ethanol; n = 7), (iii) Wortmannin (0.1 mM in 2 µl of 100 percent DMSO), (n = 7) and (ip) melatonin (4 mg/kg, dissolved in 0.9% isotonic saline/5% ethanol)/Wortmannin (0.1 mM in 2 µl of 100 percent DMSO (injected striatally)); (n = 7) immediately after reperfusion. Focal cerebral ischemia was induced using an intraluminal filament technique^11,22^. For the induction of focal cerebral ischemia, mice were anesthetized with 1% isoflurane (30% O_2_, reminder N_2_O), and rectal temperature was controlled between 36.5 and 37.0 °C using a feedback-controlled heating system. During the experiments, cerebral blood flow (CBF) was monitored via laser Doppler flowmetry (LDF) using a flexible 0.5 mm fiber optic probe (Perimed) which was attached with tissue adhesive to the intact skull overlying the MCA territory (2 mm posterior and 6 mm lateral from the bregma). After a midline neck incision, the left common and external carotid arteries were isolated and ligated. A microvascular clip (FE691; Aesculap) was temporarily placed on the internal carotid artery. A 7-0 silicon-coated nylon monofilament (701934PK5Re, Doccol) was inserted through a small incision into the common carotid artery and advanced 9 mm distal to the carotid bifurcation for MCAO. Reperfusion was initiated 30 min after the onset of ischemia by gentle monofilament removal. Thereafter, mice were placed back into their home cages. Seventy-two hours after ischemia, animals were deeply re-anesthetized and decapitated. All experimental procedures were performed in light period of the day to prevent mice from the diurnal variation.

### Neuronal survival analysis

Neuronal survival was analyzed as previously described^11^. Brain sections from mid-striatum level were fixed with 4% paraformaldehyde (PFA)/0.1 M phosphate-buffered saline (PBS) and incubated for 1 h with blocking buffer (0.1 M PBS containing 0.3% Triton X-100 (PBS-T)/10% normal goat serum (NGS)) at RT. Next, brain sections were reacted overnight with Alexa Fluor 488-conjugated monoclonal mouse anti-NeuN (Mab377X; Chemicon) at 4 °C. Following day, sections were counterstained with 4′,6-Diamidino-2-Phenylindole, Dihydrochloride (DAPI). Stained sections were analyzed under a confocal Zeiss LSM 780 microscope (Carl Zeiss). In stained sections, NeuN and DAPI positive cells were counted from 9 different region of interest (ROI), each measuring 62,500 μm^2^, in both ischemic and non-ischemic (contralateral) striatum in a blinded manner. Mean number of surviving cells were determined in the ischemic and non-ischemic striatum. Neuronal survival rate was determined by dividing the results obtained in both hemispheres.

### DNA fragmentation analysis

As previously described^11,22^, brain sections from mid-striatum level were stained by terminal transferase fluorescein-dUTP nick end labeling (TUNEL) using a *in situ* cell death detection kit (11684795910; Roche, Switzerland). Coronal brain sections were fixed with 4% PFA/0.1 M PBS and washed with PBS. Thereafter, antigen retrieval was performed by incubating the sections in citrate buffer. Then brain sections were blocked with a mixture of normal goat serum (NGS), BSA and gelatin. Lastly, they were reacted with TUNEL reaction mixture and counterstained with DAPI. In stained sections, TUNEL positive DNA-fragmented cells were counted from 9 adjacent ROI corresponding to 62,500 μm^2^ area in the striatum via LSM 780 confocal microscope (Carl Zeiss, Jena, Germany) in a blinded manner.

### Statistical analysis

For statistical data comparisons, a standard software package (SPSS 18 for Windows; SPSS Inc., Chicago, IL, USA) was used. Differences between groups were analyzed by one-way ANOVA, followed by LSD tests. All values are given as mean ± S.D. with n values, indicating the number of samples or animals analyzed. Throughout the study, p values < 0.05 were considered significant.

## Supplementary information

Supplementary Figures
