# Supplementary material for: Interaction of melatonin and Bmal1 in the regulation of PI3K/AKT pathway components and cellular survival
Source: Sci Rep. 2019 Dec 13;9:19082. doi: 10.1038/s41598-019-55663-0 (PMC6910929; doi:10.1038/s41598-019-55663-0)
Supplement: Supplementary file 1 — Supplementary Figures [file 41598_2019_55663_MOESM1_ESM.pdf]

SUPPLEMENTARY INFORMATION

Interaction of melatonin and Bmal1 in the regulation of PI3K/AKT pathway components and cellular survival

Mustafa C. Beker[1,2], Berrak Caglayan[1,3], Ahmet B. Caglayan[1,2], Taha Kelestemur[1,2], Esra Yalcin[1,2], Aysun Caglayan[1,2], Ulkan Kilic[4], Ahmet T. Baykal[1,5], Russel J. Reiter[6], Ertugrul Kilic[1,2]

[1]Regenerative and Restorative Medical Research Center, Istanbul Medipol University, 34810, Istanbul, Turkey; [2]Department of Physiology, Istanbul Medipol University, 34810, Istanbul, Turkey; [3]Department of Medical Biology, Istanbul Medipol University, 34810, Istanbul, Turkey; [4]Department of Medical Biology, Faculty of Medicine, Saglik Bilimleri University, 34668, Istanbul, Turkey; [5]Department of Medical Biochemistry, Acibadem University, 34752, Istanbul, Turkey; [6]Department of Cell Systems and Anatomy, UT Health San Antonio, 78229, Texas, USA

Running title: Role of Bmal1 in cell signaling and survival

Correspondence: Prof. Dr. Ertugrul Kilic

Department of Physiology, Istanbul Medipol University

Regenerative and Restorative Medical Research Center

Ekinciler Cad. 19, TR-34810 Istanbul, Turkey

Phone: +90-216-681 5344; Fax: +90-212-531 7555

E-mail: ekilic@medipol.edu.tr; kilic44@yahoo.com

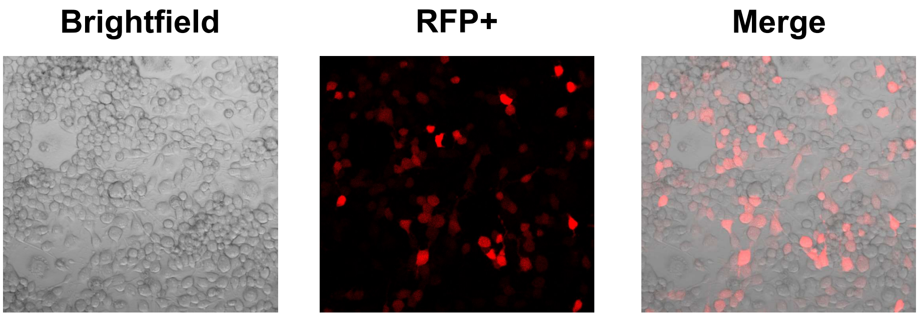

Fig. S1. Transfection efficiency of Bmal1.

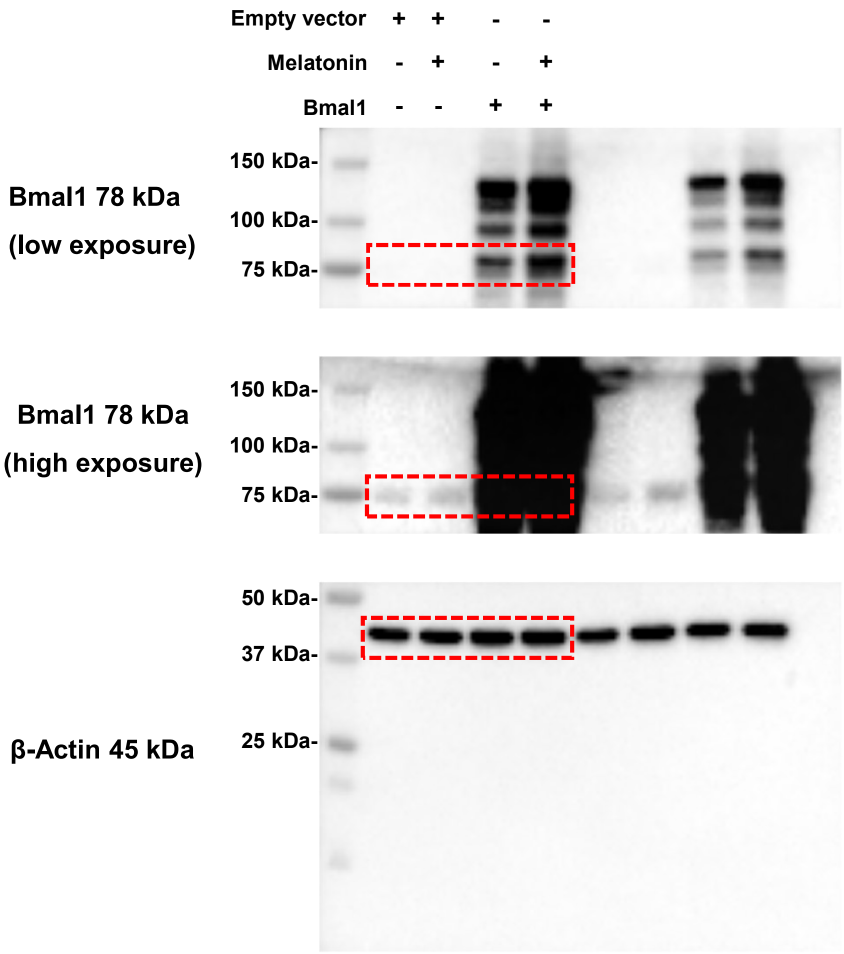

Fig. S2. Full immunoblots related to Figure 1a. These immunoblots are representative of three independent experiments. Results shown in Fig.1a are delineated by dotted rectangles. MW (in kDa) are indicated.

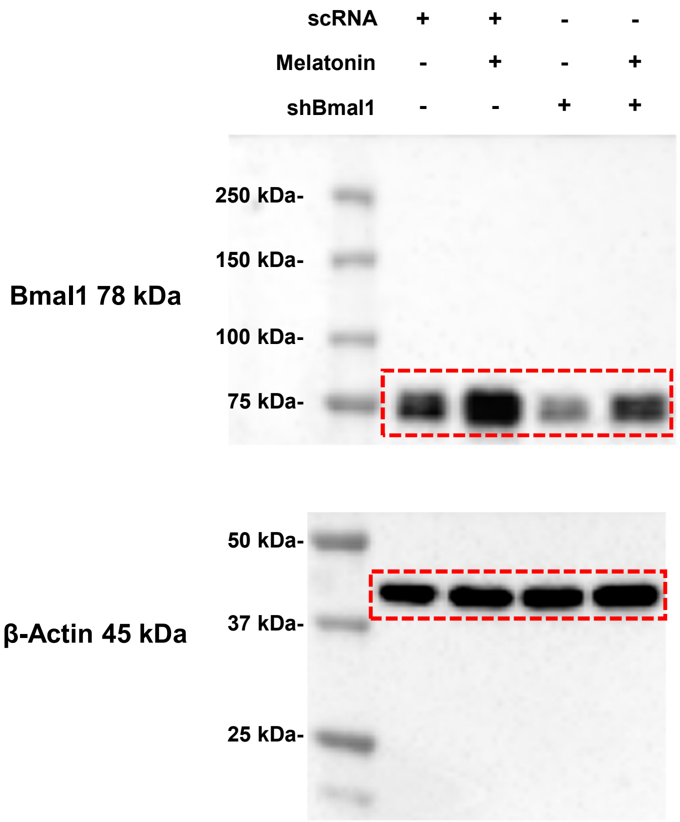

Fig. S3. Full immunoblots related to Figure 1b. These immunoblots are representative of three independent experiments. Results shown in Fig.1b are delineated by dotted rectangles. MW (in kDa) are indicated.

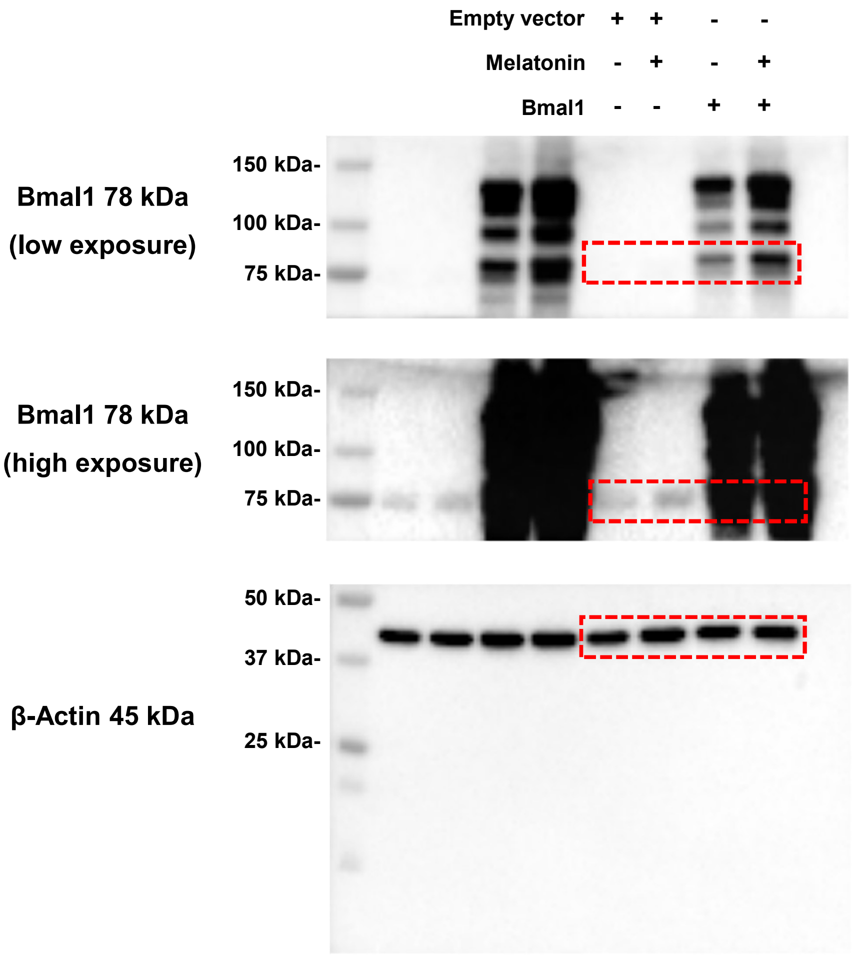

Fig. S4. Full immunoblots related to Figure 1c. These immunoblots are representative of three independent experiments. Results shown in Fig.1c are delineated by dotted rectangles. MW (in kDa) are indicated.

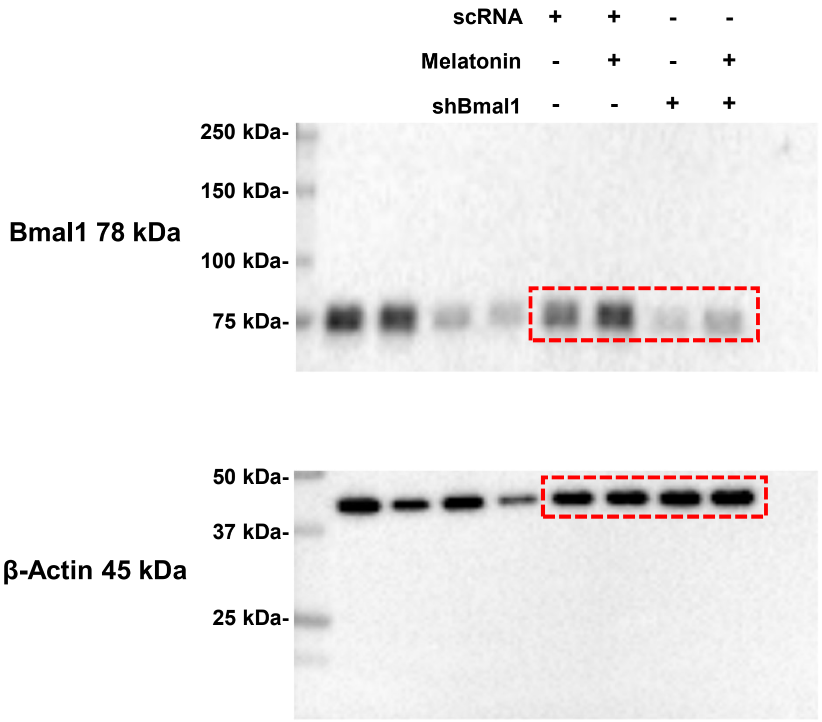

Fig. S5. Full immunoblots related to Figure 1d. These immunoblots are representative of three independent experiments. Results shown in Fig.1d are delineated by dotted rectangles. MW (in kDa) are indicated.

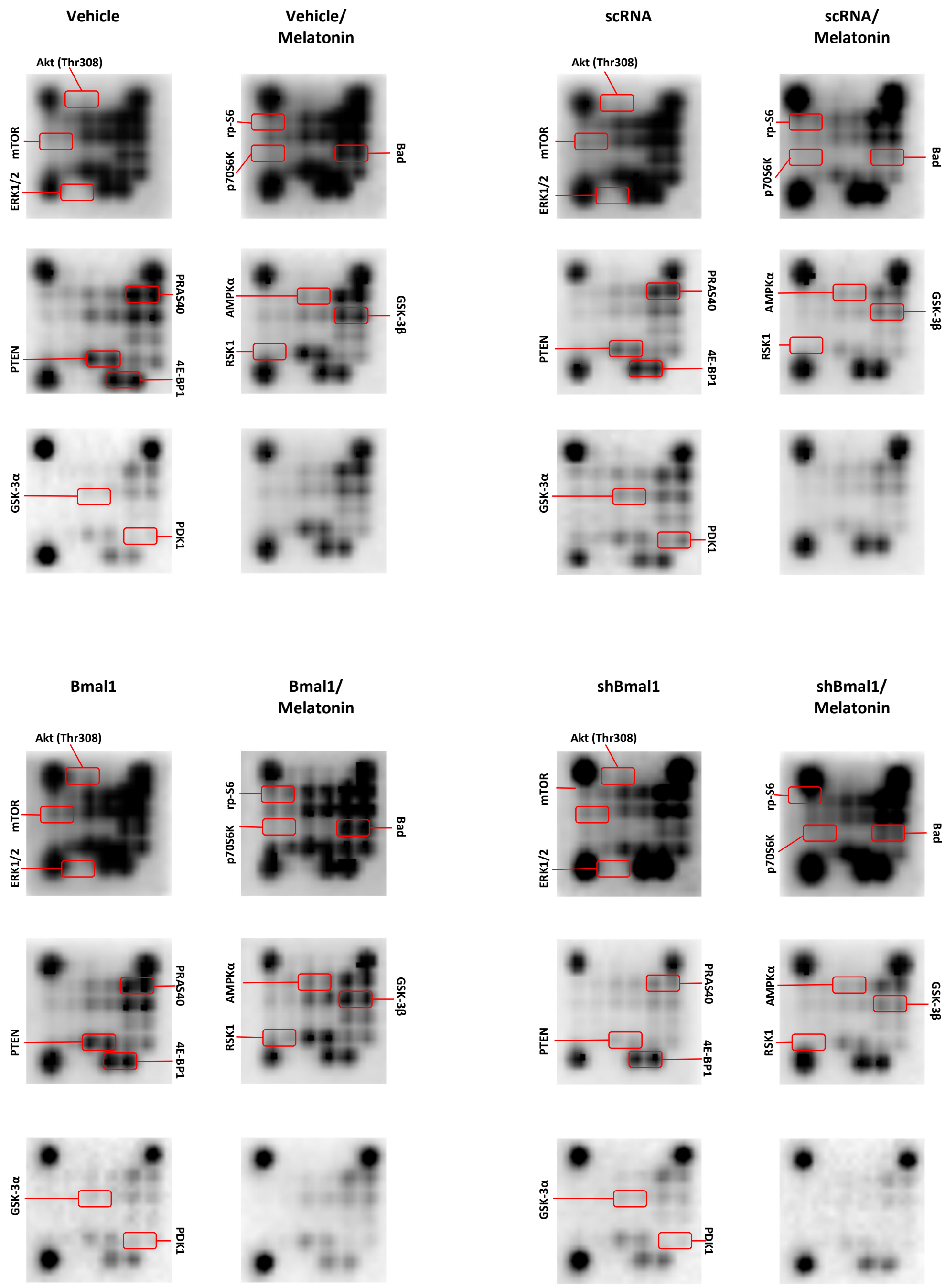

Fig. S6. Full immunoblots related to Figure 2. Results shown in Fig. 2a and Fig. 2b.

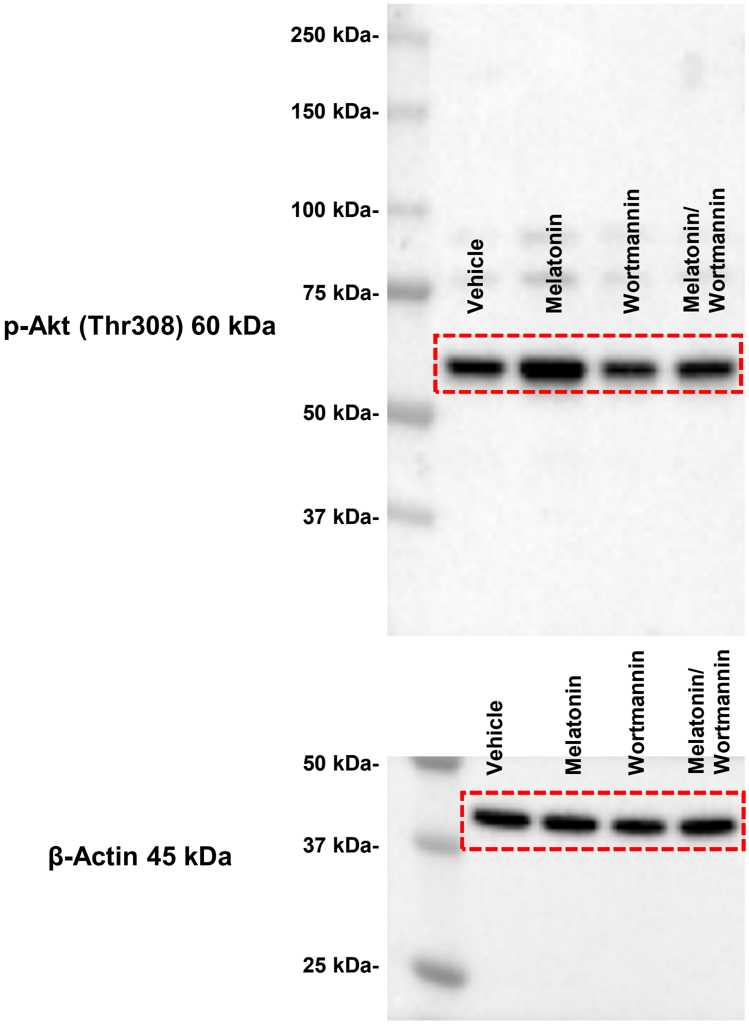

Fig. S7. Full immunoblots related to Figure 6b. These immunoblots are representative of three independent experiments. Results shown in Fig.6b are delineated by dotted rectangles. MW (in kDa) are indicated.

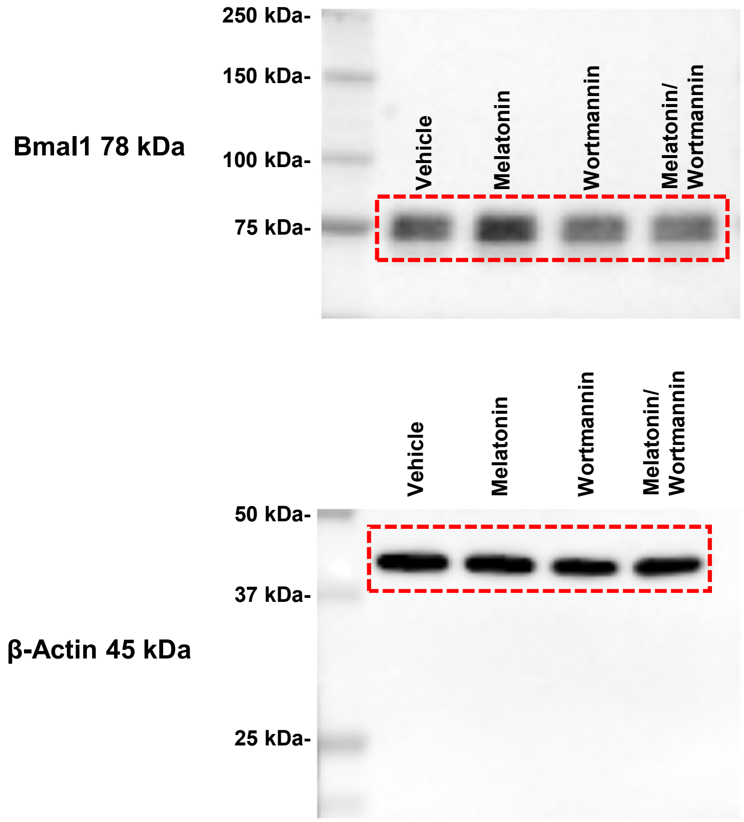

Fig. S8. Full immunoblots related to Figure 6c. These immunoblots are representative of three independent experiments. Results shown in Fig.6c are delineated by dotted rectangles. MW (in kDa) are indicated.

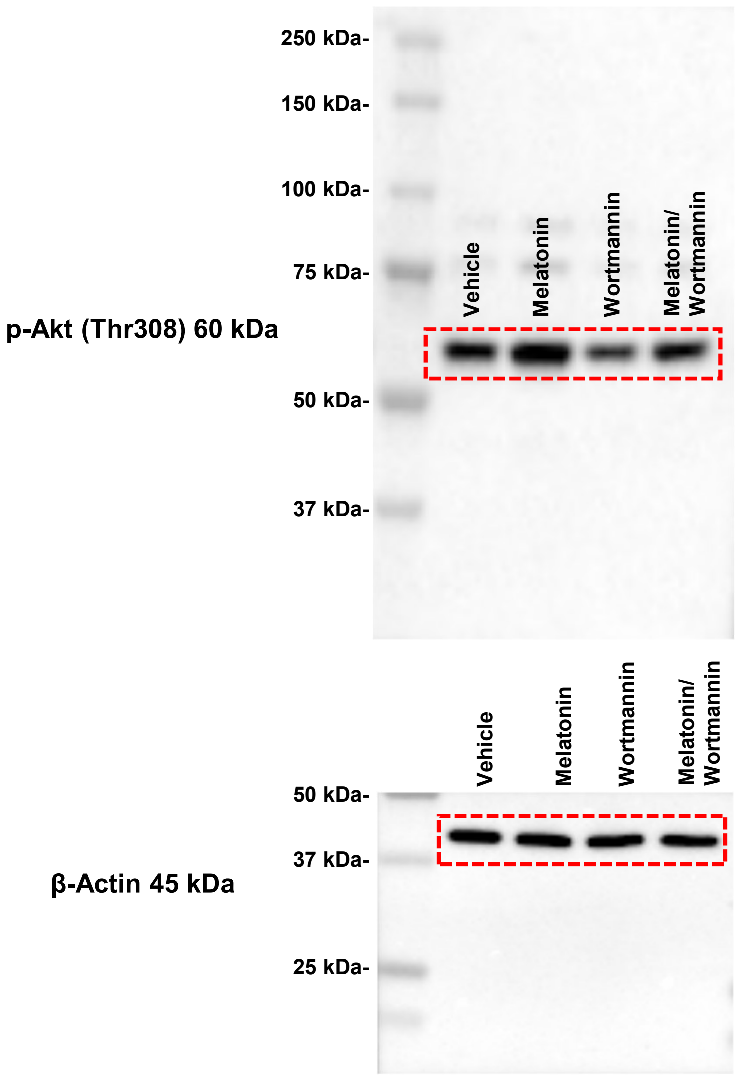

Fig. S9. Full immunoblots related to Figure 8a. These immunoblots are representative of three independent experiments. Results shown in Fig.8b are delineated by dotted rectangles. MW (in kDa) are indicated.

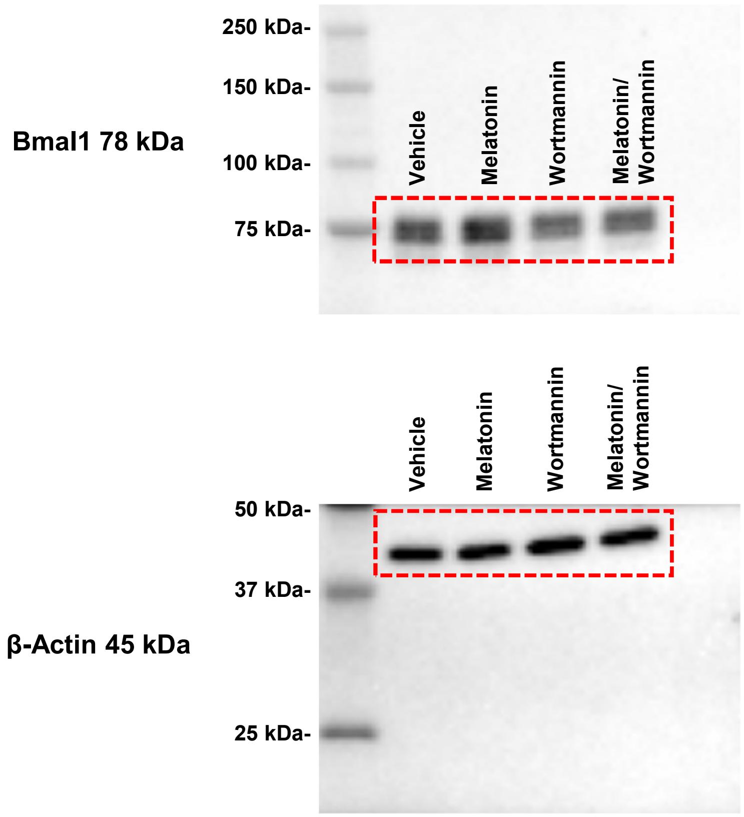

Fig. S10. Full immunoblots related to Figure 8b. These immunoblots are representative of three independent experiments. Results shown in Fig.8a are delineated by dotted rectangles. MW (in kDa) are indicated.
